# Revolutionizing breast cancer monitoring: emerging hematocrit-based metrics – a narrative review

**DOI:** 10.1097/MS9.0000000000003020

**Published:** 2025-05-21

**Authors:** Emmanuel Ifeanyi Obeagu, Getrude Uzoma Obeagu

**Affiliations:** a Department of Biomedical and Laboratory Science, Africa University, Mutare, Zimbabwe; b School of Nursing Science, Kampala International University, Ishaka, Uganda

**Keywords:** biomarkers, breast cancer, dynamic monitoring, hematocrit metrics, surveillance

## Abstract

Breast cancer remains a leading global health concern, with significant strides made in early detection and treatment. However, effective long-term surveillance, particularly for recurrence and metastasis, remains a clinical challenge. Traditional methods like imaging and biopsy are often invasive, costly, and have limited sensitivity in detecting subtle changes during disease progression. Emerging evidence suggests that hematocrit-based metrics – measurements of the proportion of red blood cells (RBCs) in blood – could serve as valuable, minimally invasive biomarkers for monitoring breast cancer. This review highlights recent advancements in hematocrit-focused research and its potential role in revolutionizing breast cancer surveillance. Hematocrit dynamics reflect complex physiological processes influenced by cancer biology, including inflammation, angiogenesis, and treatment-induced bone marrow suppression. Alterations in hematocrit levels have been associated with prognostic outcomes, treatment responses, and early indications of recurrence in breast cancer patients. Coupled with other hematological and molecular markers, hematocrit offers a cost-effective and readily accessible tool to track disease status in real time. Recent technological innovations, such as point-of-care testing, artificial intelligence-driven data analysis, and microfluidic devices, are further advancing the applicability of hematocrit-based metrics in both clinical and remote monitoring settings.

## Introduction

Breast cancer is one of the most prevalent cancers worldwide, with millions of women affected each year. While advances in early detection and therapeutic interventions have significantly improved survival rates, breast cancer remains the leading cause of cancer-related deaths among women globally. A key challenge in its management is long-term monitoring, particularly the early detection of recurrence and metastasis, which are the primary contributors to poor patient outcomes. Current surveillance methods, such as imaging and serum-based markers, have limitations in terms of sensitivity, cost, invasiveness, and accessibility. These challenges highlight the need for innovative, minimally invasive, and cost-effective tools to improve breast cancer monitoring and provide dynamic, real-time insights into disease progression and treatment response.^[1–3]^ Hematological parameters, which are routinely assessed during standard blood tests, have emerged as potential biomarkers for cancer detection and monitoring. Among these, hematocrit – the proportion of RBCs in blood – has shown promise as a novel metric for tracking disease dynamics in breast cancer. Alterations in hematocrit levels are often reflective of systemic changes induced by cancer biology, such as inflammation, angiogenesis, and treatment-related effects. As a simple, inexpensive, and widely available metric, hematocrit could complement existing surveillance approaches and provide additional layers of information for risk stratification and management^[4,5]^. In breast cancer, tumor growth and metastasis significantly impact systemic physiological processes, which in turn influence hematocrit levels. For example, cancer-induced chronic inflammation can lead to anemia of chronic disease (ACD), resulting in reduced hematocrit values. Similarly, aggressive tumor growth and angiogenesis can disrupt normal erythropoiesis and oxygen delivery, further altering hematocrit dynamics.^[6,7]^ Traditional imaging techniques, such as mammography, ultrasound, and MRI, remain the cornerstone of breast cancer detection and follow-up. However, these methods have notable drawbacks, including high costs, limited access in low-resource settings, and their inability to capture subtle systemic changes that precede overt clinical manifestations. Biopsy, another critical tool in breast cancer diagnosis, is invasive and not feasible for frequent monitoring. Serum biomarkers such as CA 15-3 and carcinoembryonic antigen are widely used but often lack sensitivity and specificity, particularly in the early stages of recurrence. Hematocrit-based metrics, in contrast, offer a less invasive and more dynamic approach, reflecting systemic changes in real time^[8,9]^. Beyond its diagnostic potential, hematocrit could play an essential role in monitoring treatment response. Chemotherapy, hormonal therapy, and radiation therapy often lead to hematological side effects, such as anemia or bone marrow suppression, which can be tracked through hematocrit levels. Trends in hematocrit during therapy may provide valuable insights into treatment efficacy and patient tolerability, enabling personalized adjustments to therapeutic regimens. Moreover, stable or improving hematocrit levels during treatment could indicate reduced tumor burden and improved systemic health, while persistent anemia might signal suboptimal response or emerging complications^[10,11]^. The integration of hematocrit into breast cancer surveillance has been further propelled by advancements in technology. Point-of-care (POC) devices now enable rapid and accurate hematocrit measurement outside of traditional laboratory settings, making it feasible to track changes even in remote or resource-limited environments. Artificial intelligence (AI) and machine learning (ML) algorithms are also being developed to analyze trends in hematocrit alongside other clinical and molecular data, enhancing the predictive power of this metric. These innovations highlight the potential of hematocrit to evolve from a static measurement into a dynamic biomarker with clinical relevance across diverse healthcare settings^[12,13]^.

**Highlights**

- Hematocrit metrics beyond anemia: Emerging research shows hematocrit’s potential in monitoring breast cancer progression.
- Innovations in measurement techniques: Advances like point-of-care devices and automated analyzers offer new opportunities.
- Complementary to existing biomarkers: Hematocrit metrics can be used alongside current breast cancer biomarkers.
- Challenges and limitations identified: Key challenges include standardizing measurement techniques, improving sensitivity and specificity, and integrating hematocrit metrics with other biomarkers.
- Future research opportunities: Future studies should focus on developing standardized protocols.

### Aim

The aim of this review is to critically evaluate recent advancements in hematocrit-based biomarkers and explore their clinical utility in the context of breast cancer management.

### Rationale of the review

Breast cancer surveillance remains a cornerstone of patient management, as early detection of recurrence or metastasis significantly improves outcomes. Despite substantial progress in diagnostic and treatment modalities, existing monitoring tools such as imaging and serum-based markers have notable limitations, including invasiveness, high costs, and a lack of sensitivity in detecting early systemic changes. These limitations underscore the urgent need for innovative approaches that are noninvasive, accessible, and capable of providing dynamic, real-time insights into disease progression. Hematocrit, a routine hematological parameter reflecting the proportion of RBCs in blood, has recently garnered attention as a potential biomarker for cancer monitoring. Its utility in breast cancer, however, remains underexplored^[1,2]^. Hematocrit is an easily obtainable metric, measured in routine blood tests, making it a cost-effective and widely accessible tool for longitudinal monitoring. Its ability to reflect systemic physiological changes – such as those induced by tumor biology, chronic inflammation, or treatment effects – positions it as a promising biomarker for breast cancer surveillance. Emerging evidence links alterations in hematocrit levels with cancer progression, prognosis, and treatment response, suggesting its potential to complement existing diagnostic and monitoring strategies. Furthermore, technological advancements, such as POC testing devices and AI-driven analysis, have created opportunities to harness hematocrit in new and innovative ways.^[3–5]^ This review is motivated by the growing interest in hematological parameters as minimally invasive cancer biomarkers and the need to better understand hematocrit’s role in breast cancer surveillance. By synthesizing current evidence and highlighting recent innovations, this review seeks to explore the potential of hematocrit-based metrics to address existing gaps in breast cancer monitoring. Additionally, it aims to identify challenges and propose future research directions to guide the integration of hematocrit into clinical practice. Ultimately, this review aspires to contribute to the ongoing quest for more effective and patient-centered approaches to breast cancer care.

## Review methodology

This report outlines the methodology used for conducting a narrative review of recent advances in hematocrit-based biomarkers for breast cancer surveillance. A well-structured review methodology ensures the comprehensive inclusion of relevant studies and provides a clear, systematic approach to analyzing and summarizing existing research in the field. The following methodology was adopted to achieve these objectives.

### Literature search strategy

A systematic and comprehensive search strategy was employed to gather relevant articles. The following databases were searched:

- PubMed
- Google Scholar
- Scopus
- Web of Science
- Embase

The search terms included combinations of the following keywords:

- “Hematocrit”
- “Breast cancer”
- “Surveillance”
- “Anemia”
- “Biomarkers”
- “Tumor hypoxia”
- “Treatment response”
- “Point-of-care testing”

The search was limited to peer-reviewed articles published between 2010 and 2024. This time frame was chosen to capture the most recent advances in the field while ensuring relevance to contemporary clinical practices.

### Inclusion and exclusion criteria

To ensure the quality and relevance of the selected studies, the following inclusion and exclusion criteria were applied:

Inclusion criteria:

- Studies focused on breast cancer surveillance using hematocrit-based biomarkers.
- Research articles that examined the role of hematocrit in assessing treatment response, tumor oxygenation, or inflammation in breast cancer patients.
- Clinical trials, systematic reviews, meta-analyses, and observational studies that reported on the use of hematocrit for monitoring breast cancer.

Exclusion criteria:

- Studies not related to breast cancer or hematocrit-based biomarkers.
- Animal studies, as the review aimed to focus on human clinical research.
- Articles not published in English or lacking full-text access.

### Hematocrit dynamics in breast cancer biology

Hematocrit, a measure of the proportion of RBCs in the blood, is closely linked to systemic physiological processes and is often altered in cancer patients. In breast cancer, hematocrit levels are influenced by complex interactions between the tumor microenvironment (TME), systemic inflammation, and the hematopoietic system. These changes are driven by both the direct effects of cancer biology and indirect effects related to treatment interventions[14]. One of the primary ways breast cancer impacts hematocrit is through chronic inflammation. Tumors induce an inflammatory response by releasing cytokines, such as interleukin-6 (IL-6) and tumor necrosis factor-alpha (TNF-α), which disrupt normal erythropoiesis and iron metabolism. These cytokines promote the development of ACD, characterized by reduced hematocrit levels due to impaired RBC production and decreased iron availability. This systemic inflammatory state is further exacerbated by the immune response to the tumor, which creates a feedback loop that perpetuates anemia in cancer patients. Thus, low hematocrit levels are often observed in breast cancer patients, particularly in those with advanced or metastatic disease[15]. Angiogenesis, a hallmark of cancer progression, also plays a role in hematocrit dynamics. Breast tumors stimulate the formation of abnormal blood vessels to meet their oxygen and nutrient demands. This vascular remodeling can disrupt the oxygen supply to the bone marrow, impairing erythropoiesis and contributing to anemia. In addition, the high metabolic activity of tumors often leads to increased systemic hypoxia, which can paradoxically trigger erythropoietin (EPO) production in an attempt to compensate for oxygen deficits. This compensatory response, however, is often insufficient to counteract the effects of inflammation and impaired marrow function, leading to further hematocrit fluctuations[16]. The hematological effects of breast cancer treatments also influence hematocrit levels. Chemotherapy, one of the most common treatments for breast cancer, directly suppresses bone marrow activity, reducing the production of RBCs. This cytotoxic effect often leads to treatment-induced anemia, particularly in regimens involving platinum-based or anthracycline drugs. Radiation therapy targeting the chest or breast area can similarly damage the bone marrow, exacerbating hematocrit decline. Hormonal therapies, such as aromatase inhibitors or selective estrogen receptor (ER) modulators, may also indirectly affect hematocrit by altering systemic metabolism and inflammation. These treatment-induced changes in hematocrit offer a valuable opportunity to monitor therapy response and side effects[10]. Hematocrit dynamics are also influenced by nutritional deficiencies, a common issue in breast cancer patients. Malnutrition, reduced appetite, or side effects from treatment can result in deficiencies in iron, vitamin B12, and folate – key nutrients required for erythropoiesis. These deficiencies further exacerbate anemia and hematocrit decline. Additionally, the psychosocial burden of cancer, including stress and depression, can impair overall health and contribute to hematological imbalances. Monitoring hematocrit levels in the context of these broader systemic changes can provide a holistic view of patient health[17]. Moreover, emerging evidence suggests that hematocrit levels may reflect the metastatic potential of breast cancer. Studies indicate that circulating tumor cells (CTCs) can interact with erythrocytes, altering blood viscosity and hematocrit dynamics. In advanced stages of the disease, these interactions may contribute to microvascular occlusion and localized hypoxia, further disrupting hematological homeostasis. While the exact mechanisms remain under investigation, these findings underscore the potential utility of hematocrit as an indicator of disease progression and metastasis[18]. In addition to its biological underpinnings, hematocrit dynamics may have prognostic significance in breast cancer. Several studies have reported that lower hematocrit levels at diagnosis are associated with poorer overall survival and higher recurrence rates. This association likely reflects the systemic impact of aggressive tumors on the hematopoietic system and overall patient health. Conversely, stable or improving hematocrit levels during treatment may serve as a positive prognostic indicator, reflecting effective tumor control and recovery of systemic function[19]. The dynamic nature of hematocrit, influenced by both cancer biology and treatment effects, makes it an attractive candidate for integration into breast cancer surveillance. As a marker of systemic changes, hematocrit provides information beyond localized tumor characteristics, offering a broader perspective on patient health. However, its sensitivity to various non-cancer-related factors, such as hydration status, altitude, and comorbid conditions, underscores the need for careful interpretation and integration with other biomarkers[20]. Hematocrit’s role in breast cancer biology extends beyond a simple measurement of RBC volume; it is a window into the systemic effects of cancer and its treatment. By leveraging its accessibility and dynamic nature, hematocrit has the potential to complement existing surveillance tools and improve patient outcomes. Further research into the mechanistic links between hematocrit and cancer progression will help refine its application as a biomarker, paving the way for its integration into personalized breast cancer care[21].

### Hematocrit metrics and their relevance to breast cancer

The relationship between hematocrit levels and breast cancer progression stems from the systemic physiological changes induced by the disease, including inflammation, tumor angiogenesis, and treatment-related side effects. By reflecting these changes, hematocrit metrics hold promise as a dynamic, accessible biomarker for various stages of breast cancer management[14].

### The connection between hematocrit and cancer biology

In breast cancer, systemic inflammation driven by tumor-secreted cytokines, such as IL-6 and TNF-α, disrupts normal erythropoiesis and iron metabolism. This results in ACD, characterized by reduced hematocrit levels. Tumor growth and angiogenesis further contribute to hypoxia and vascular remodeling, impairing oxygen delivery and bone marrow function. These physiological disruptions make hematocrit a valuable indicator of the systemic effects of breast cancer, as its fluctuations can signal disease progression or response to treatment[15].

### Hematocrit as a prognostic indicator

Hematocrit levels have been associated with breast cancer prognosis, with lower levels at diagnosis often correlating with advanced disease and poorer outcomes. Anemia, reflected by reduced hematocrit, is a hallmark of aggressive cancers due to the inflammatory and metabolic demands imposed by rapidly growing tumors. Patients with low hematocrit may also experience more severe fatigue and reduced functional status, factors that can negatively influence treatment outcomes and overall survival. Hematocrit’s role as a prognostic marker highlights its potential utility in stratifying patients into risk categories at the time of diagnosis[16].

### Hematocrit and treatment response

During treatment, hematocrit levels can serve as a dynamic marker for monitoring therapeutic effects and patient well-being. Chemotherapy, a standard treatment for breast cancer, often suppresses bone marrow function, leading to anemia and declining hematocrit levels. Radiation therapy and hormonal treatments may also indirectly affect hematopoiesis, further influencing hematocrit values. Tracking hematocrit levels over the course of treatment can provide insights into both the efficacy and tolerability of therapy. For example, improving hematocrit levels may indicate effective tumor control and recovery of systemic function, while persistent anemia could suggest treatment failure, toxicity, or underlying complications[10].

### Hematocrit and recurrence monitoring

Detecting breast cancer recurrence remains a critical challenge, as early signs are often subtle and systemic. Hematocrit metrics, when combined with other hematological markers, can provide early warning signals for recurrence. For instance, declining hematocrit levels in a patient previously stable after treatment could indicate emerging inflammation or metastatic activity. Studies suggest that hematocrit, along with markers like neutrophil-to-lymphocyte ratio, can enhance the sensitivity of recurrence surveillance protocols, offering a less invasive and more cost-effective alternative to imaging or biopsies[17].

### Integration with multimodal biomarker models

Hematocrit metrics are unlikely to replace established breast cancer biomarkers, such as CTCs or cell-free DNA. Instead, they can complement these markers by providing systemic, real-time insights into patient health. By integrating hematocrit data into multimodal biomarker panels, clinicians can improve diagnostic accuracy and gain a more comprehensive view of disease progression and treatment response. For example, hematocrit trends could be analyzed alongside molecular and imaging data to refine patient management strategies and improve outcomes[18].

### Hematocrit as a measure of patient quality of life

Beyond its role in disease monitoring, hematocrit is also relevant to patient-reported outcomes and quality of life (QoL). Low hematocrit levels are often associated with symptoms such as fatigue, reduced physical performance, and impaired cognitive function. These symptoms can significantly affect the QoL for breast cancer patients, particularly during treatment. By monitoring hematocrit trends, clinicians can identify patients at risk for anemia-related complications and implement supportive measures, such as nutritional interventions or erythropoiesis-stimulating agents, to improve QoL[18].

### Mechanisms underlying hematocrit changes in breast cancer

Hematocrit, a measure of the proportion of RBCs in blood, undergoes significant alterations in breast cancer patients. These changes reflect a complex interplay of cancer biology, systemic responses, and treatment effects[19].

#### Tumor-induced inflammation and hematopoiesis suppression

Breast cancer often induces a systemic inflammatory response characterized by elevated levels of cytokines such as IL-6 and TNF-α. These cytokines suppress erythropoiesis in the bone marrow, leading to a reduction in hematocrit levels. Persistent inflammation alters iron metabolism by increasing hepcidin production, which sequesters iron in macrophages and reduces its availability for erythropoiesis. This mechanism is a primary contributor to lower hematocrit levels in breast cancer patients^[20,21]^.

#### Hypoxia and EPO dysregulation

Rapid tumor growth often outpaces its blood supply, leading to localized hypoxia. In response, hypoxia-inducible factors stimulate EPO production, which can temporarily increase RBC production and hematocrit. Some breast cancer subtypes may directly secrete EPO or other growth factors that stimulate erythropoiesis, causing an abnormal rise in hematocrit. However, these effects are often offset by systemic inflammation and myelosuppression^[22,23]^.

#### Bone marrow involvement and myelosuppression

Advanced breast cancer with metastasis to the bone marrow disrupts normal hematopoiesis, leading to pancytopenia and decreased hematocrit. Common breast cancer treatments, such as anthracyclines and taxanes, target rapidly dividing cells, including erythroid precursors in the bone marrow. This results in anemia and a corresponding drop in hematocrit levels^[24,25]^.

#### Angiogenesis and vascular permeability

Breast tumors promote the formation of abnormal, leaky blood vessels to support their growth. Increased vascular permeability causes plasma leakage into the surrounding tissue, leading to hemoconcentration and transient changes in hematocrit. Breast cancer-related complications, such as lymphatic obstruction, can lead to localized or systemic fluid retention. This dilutes blood components, causing apparent decreases in hematocrit^[25–27]^.

#### Nutritional deficiencies and cachexia

Breast cancer patients, especially those with advanced disease, often experience deficiencies in iron, vitamin B12, and folate due to poor nutritional intake or malabsorption. These deficiencies impair RBC production and reduce hematocrit. A syndrome of metabolic dysfunction and muscle wasting in advanced cancer stages exacerbates anemia by diminishing the body’s ability to produce and sustain RBCs^[28,29]^.

#### Treatment-related mechanisms

Localized or systemic radiation can damage bone marrow, further suppressing erythropoiesis and reducing hematocrit. Novel treatments may inadvertently affect hematological parameters. For example, immune checkpoint inhibitors can exacerbate autoimmune phenomena, leading to hemolysis or marrow suppression[30].

#### Hemolysis and blood loss

In rare cases, breast cancer therapies or autoimmune processes associated with malignancy can lead to RBC destruction, decreasing hematocrit. Surgical interventions, biopsies, or gastrointestinal bleeding due to cancer-related complications may result in acute or chronic reductions in hematocrit[31].

#### Systemic effects of comorbidities

Breast cancer patients with renal comorbidities may exhibit reduced EPO production, contributing to anemia and lower hematocrit. Treatments such as anthracyclines, which can cause cardiotoxicity, indirectly influence hematocrit by affecting fluid balance and EPO regulation[32].

### Angiogenesis-promoting cytokines in breast cancer

Angiogenesis, the process of new blood vessel formation, is a hallmark of cancer progression, including breast cancer. Tumor-induced angiogenesis is driven by a complex network of cytokines and growth factors that orchestrate the proliferation, migration, and differentiation of endothelial cells. These molecules not only sustain tumor growth by ensuring an adequate blood supply but also facilitate metastasis and immune evasion[19].

#### Key angiogenesis-promoting cytokines in breast cancer

##### Vascular endothelial growth factor (VEGF)

VEGF is the primary mediator of angiogenesis in breast cancer. It promotes endothelial cell proliferation, migration, and survival while increasing vascular permeability. VEGF binds to its receptors (VEGFR-1, VEGFR-2) on endothelial cells, activating downstream signaling pathways like PI3K/Akt and MAPK, leading to angiogenesis. Elevated VEGF levels are associated with aggressive tumor phenotypes and poor prognosis in breast cancer patients[20].

##### Platelet-derived growth factor (PDGF)

PDGF supports pericyte recruitment and vascular maturation in newly formed blood vessels, stabilizing the angiogenic network. PDGF interacts with PDGFR-β on stromal and endothelial cells, enhancing vessel integrity and sustaining tumor vasculature. PDGF signaling is a target for anti-angiogenic therapies in breast cancer[21].

##### Fibroblast growth factor (FGF)

FGF, particularly FGF-2 (basic FGF), stimulates endothelial cell proliferation and differentiation, contributing to sustained angiogenesis. FGF binds to FGFR, activating pathways like Ras-MAPK and PI3K-Akt, which drive endothelial cell growth and survival. Aberrant FGF signaling correlates with resistance to certain anti-angiogenic treatments[22].

##### Interleukin-8 (IL-8)

IL-8 acts as a pro-angiogenic cytokine by enhancing endothelial cell migration and capillary tube formation. IL-8 binds to CXCR1 and CXCR2 receptors, activating pathways such as NF-κB and ERK1/2, promoting angiogenesis and tumor cell survival. High IL-8 levels are linked to increased breast cancer invasiveness and poor therapeutic response[23].

##### TNF-α

TNF-α indirectly promotes angiogenesis by upregulating VEGF and FGF expression in the TME. TNF-α induces NF-κB activation in tumor and stromal cells, enhancing the expression of pro-angiogenic mediators. Paradoxically, while TNF-α promotes angiogenesis at low concentrations, high levels may exert anti-angiogenic effects[24].

##### Transforming growth factor-beta (TGF-β)

TGF-β has dual roles in angiogenesis, depending on the tumor stage. It can enhance angiogenesis in advanced tumors by promoting VEGF and connective tissue growth factor expression. TGF-β activates Smad-dependent and Smad-independent pathways, influencing endothelial cell behavior and extracellular matrix (ECM) remodeling. Elevated TGF-β levels are often found in metastatic breast cancer[25].

##### Angiopoietins (Ang-1 and Ang-2)

These cytokines modulate vessel stability and remodeling. Ang-2, in particular, disrupts vascular stability, promoting VEGF-driven angiogenesis. Ang-1 and Ang-2 interact with the Tie2 receptor on endothelial cells, regulating vascular permeability and sprouting. The Ang-2/Tie2 axis is a potential therapeutic target in breast cancer angiogenesis[26].

#### Interaction with the TME

Cancer-associated fibroblasts secrete VEGF, FGF, and PDGF, supporting angiogenesis. Tumor-associated macrophages release VEGF, IL-8, and TNF-α, amplifying the pro-angiogenic milieu. ECM remodeling by matrix metalloproteinases facilitates cytokine release and endothelial cell migration[27].

##### Cellular landscape of hematocrit metrics for dynamic breast cancer surveillance

Breast cancer remains one of the leading causes of morbidity and mortality worldwide, necessitating advancements in early detection and monitoring techniques for better patient management. Traditional surveillance methods such as mammography, ultrasound, and biopsy, while effective, often have limitations in terms of sensitivity, specificity, and invasiveness. The growing interest in hematocrit metrics as a tool for dynamic surveillance in breast cancer offers a noninvasive alternative that could significantly improve detection and monitoring of the disease[28]. Hematocrit (Hct) is a critical blood marker that reflects the proportion of RBCs in the blood. It is a direct indicator of the blood’s ability to carry oxygen and is often used to diagnose conditions such as anemia or polycythemia. However, emerging evidence suggests that hematocrit levels and their dynamics could offer valuable insights into cancer progression, especially in breast cancer. Changes in hematocrit could reflect a variety of physiological responses to cancer, such as tumor-induced hypoxia, the systemic inflammatory response, or bone marrow adaptation to disease[29]. The TME is a complex ecosystem that consists of tumor cells, immune cells, blood vessels, fibroblasts, and ECM components. Hypoxia, a hallmark of many solid tumors, plays a central role in shaping the TME. Tumor hypoxia leads to changes in local blood flow, affecting hematocrit levels. As tumors grow, they often outpace their blood supply, resulting in regions of low oxygen (hypoxic zones), which can trigger the release of VEGF and other angiogenic factors. These changes not only alter the local microvasculature but also influence systemic blood parameters, including hematocrit. Thus, variations in hematocrit levels could serve as an indirect biomarker of tumor activity and response to therapy[30]. Several studies have indicated that hematocrit levels may be associated with breast cancer prognosis. For instance, lower hematocrit levels have been observed in patients with advanced-stage breast cancer, possibly reflecting increased tumor burden, anemia due to chronic disease, or bone marrow suppression. Conversely, a rise in hematocrit could suggest dehydration, stress, or response to chemotherapy. The ability to dynamically track hematocrit changes over time provides a tool for monitoring disease progression, assessing treatment efficacy, and identifying complications such as tumor-induced anemia or polycythemia[31]. Hematocrit dynamics have been shown to correlate with chemotherapy response in breast cancer patients. Chemotherapy can affect hematopoiesis, often leading to a reduction in RBC production and, consequently, a decrease in hematocrit levels. Monitoring these levels throughout treatment can offer clinicians insights into the hematologic side effects of chemotherapy, such as anemia, and help adjust treatment regimens accordingly. Additionally, understanding the kinetics of hematocrit changes during chemotherapy cycles could assist in distinguishing between therapeutic effects and disease progression[32]. The inflammatory response plays a key role in the pathogenesis and progression of breast cancer. Hematocrit levels can be affected by systemic inflammation, which is often present in cancer patients. Inflammatory cytokines, such as interleukins and TNF-α, can influence RBC production and survival, leading to changes in hematocrit. Thus, tracking hematocrit as part of a broader immune response profile could provide valuable information about disease progression and therapeutic effectiveness, particularly in the context of targeted or immunotherapy-based treatments[33]. Dynamic breast cancer surveillance refers to the continuous monitoring of various biomarkers and physiological parameters to track changes in disease status over time. Integrating hematocrit measurements into such surveillance protocols could significantly enhance the precision and personalization of breast cancer management. Regular tracking of hematocrit levels, alongside other biomarkers such as tumor markers and imaging techniques, would allow for a more comprehensive view of a patient’s health status. This could aid in the early detection of recurrence, provide a better understanding of therapy responses, and enable more timely interventions[34].

## Markers for inflammation and hematocrit metrics in dynamic breast cancer surveillance

### Inflammatory markers in breast cancer

Inflammation plays a crucial role in the initiation, progression, and metastasis of cancer, including breast cancer. It is increasingly recognized that the TME, shaped by inflammatory cells, cytokines, and immune responses, significantly contributes to tumor development. Several inflammatory markers have been studied as potential indicators for monitoring breast cancer:

1. C-reactive protein (CRP): CRP is a well-known acute-phase reactant produced by the liver in response to inflammation. Elevated CRP levels have been associated with poor prognosis in breast cancer, indicating higher tumor burden, lymph node involvement, and metastatic disease. Regular monitoring of CRP levels in breast cancer patients may assist in evaluating treatment efficacy and detecting recurrence[35].
2. Interleukins (IL-6, IL-1β): These cytokines are involved in both inflammation and immune responses. IL-6, in particular, has been linked to cancer progression through its role in promoting tumor growth, angiogenesis, and immune evasion. Elevated levels of IL-6 and IL-1β have been detected in breast cancer patients, correlating with advanced disease stages and poorer outcomes[36].
3. TNF-α: TNF-α is a potent pro-inflammatory cytokine involved in immune cell recruitment and the promotion of inflammatory responses. In breast cancer, TNF-α is often elevated and may influence tumor survival and metastasis. Targeting TNF-α has been explored as a therapeutic strategy in breast cancer treatment.
4. Fibrinogen: Elevated fibrinogen levels are often observed in cancer patients and correlate with disease severity and poor prognosis. As a marker of systemic inflammation, fibrinogen can provide real-time insights into the inflammatory status of breast cancer patients[37].

### Hematocrit metrics and dynamic surveillance

Hematocrit, the percentage of RBCs in blood, has traditionally been a marker of blood volume and oxygen-carrying capacity. However, emerging evidence suggests that hematocrit may have more nuanced implications in cancer surveillance, especially in breast cancer.

1. Changes in Hematocrit as a response to inflammation: Inflammation can affect hematocrit levels, with chronic inflammatory conditions often leading to ACD. A drop in hematocrit could indicate a systemic inflammatory response or bone marrow suppression, often seen in advanced cancer or as a side effect of chemotherapy. Dynamic monitoring of hematocrit levels can thus provide valuable information about a patient’s inflammatory status and response to treatment[38].
2. Anemia and breast cancer prognosis: Anemia, commonly associated with low hematocrit, is a frequent finding in breast cancer patients, particularly those undergoing chemotherapy or with advanced disease. Anemia negatively impacts QoL and may indicate poor prognosis. Monitoring hematocrit as part of dynamic surveillance can help identify anemia early and guide therapeutic interventions, such as erythropoiesis-stimulating agents or blood transfusions[39].
3. Hematocrit as a potential marker for chemotherapy-induced toxicity: Chemotherapy-induced bone marrow suppression is a well-known complication, often leading to decreased hematocrit levels. Monitoring these metrics during chemotherapy can help predict and manage the risk of hematologic toxicity. Adjustments to treatment protocols or supportive care measures can be made in real-time based on hematocrit trends[40].
4. Hematocrit and tumor oxygenation: Low hematocrit levels can result in reduced oxygen delivery to tissues, including tumors. Tumor hypoxia is a critical factor influencing tumor growth, metastasis, and response to therapy. Therefore, changes in hematocrit levels can indirectly reflect tumor oxygenation status, which can influence treatment planning, particularly for therapies targeting hypoxic tumors[41].

## The integration of inflammatory and hematocrit metrics in surveillance

Combining inflammatory markers and hematocrit metrics offers a comprehensive approach to dynamic breast cancer surveillance. For instance, a rise in inflammatory markers coupled with a drop in hematocrit could signal disease progression, while stable levels may suggest effective treatment control. This dual approach enhances early detection of recurrence, allows for tailored treatment strategies, and improves patient outcomes by providing real-time insights into both the systemic and localized effects of breast cancer. Moreover, advanced technologies, such as digital health tools and biomarker monitoring platforms, can facilitate the integration of these markers into clinical practice, ensuring more personalized and responsive breast cancer care[9].

## Clinical utility of hematocrit metrics in breast cancer

The clinical utility of hematocrit metrics in breast cancer represents a promising avenue for enhancing patient management through noninvasive, cost-effective monitoring. Hematocrit, which measures the proportion of blood volume occupied by RBCs, serves as a valuable biomarker for assessing disease status, guiding treatment decisions, and monitoring therapeutic responses in breast cancer patients. This section explores the various ways in which hematocrit metrics can be leveraged in clinical practice to improve breast cancer care.

### Noninvasive monitoring of disease progression

One of the primary benefits of hematocrit metrics is their noninvasive nature[33]. Unlike imaging modalities such as mammography or MRI, which require specialized equipment and can be uncomfortable for patients, blood tests for hematocrit are quick, minimally invasive, and low-cost. This allows for frequent monitoring of patients without causing additional physical or financial burdens. Regular assessment of hematocrit levels can provide real-time insights into disease progression and help detect changes in tumor status between scheduled imaging sessions. For example, a significant drop in hematocrit levels might indicate the development of anemia associated with advanced disease or an adverse reaction to therapy, prompting further investigation or intervention.

### Complementing existing diagnostic methods

Hematocrit metrics can complement existing diagnostic methods, such as imaging and tissue biopsies, by providing additional layers of information[34]. While imaging techniques are essential for visualizing tumors and detecting metastasis, they have limitations in terms of sensitivity and specificity. Tissue biopsies, though definitive for diagnosis, are invasive and not suitable for frequent use. Hematocrit metrics offer a less invasive means to assess disease status and monitor changes over time. For instance, combined analysis of hematocrit levels with imaging results can provide a more comprehensive picture of the tumor’s impact on the patient’s overall health and response to treatment.

### Guiding treatment decisions

Hematocrit metrics can assist in guiding treatment decisions throughout the course of breast cancer management[35]. Changes in hematocrit levels can reflect the effectiveness of ongoing treatments, such as chemotherapy, targeted therapies, or hormonal therapies. A decrease in hematocrit levels during treatment may signal that the patient is experiencing side effects such as anemia, which may require adjustment of the treatment regimen or the addition of supportive care measures like erythropoiesis-stimulating agents or iron supplements. Conversely, stable or improving hematocrit levels might indicate that the treatment is effective and that the patient is responding well. This use of hematocrit metrics helps clinicians tailor therapies to individual patient needs and manage treatment-related adverse effects.

### Detecting anemia and its underlying causes

Anemia is a common complication in breast cancer patients, often resulting from the disease itself or its treatments[36]. Hematocrit metrics are central to diagnosing and managing anemia, providing a straightforward measure of RBC volume. When anemia is detected, further investigation is required to determine its cause, which may include chronic disease, nutritional deficiencies, or bone marrow suppression. By monitoring hematocrit levels, clinicians can differentiate between anemia caused by the cancer’s systemic effects and anemia resulting from treatment side effects. This distinction is crucial for selecting appropriate therapeutic strategies, such as iron supplementation, vitamin therapy, or adjusting cancer treatments to mitigate adverse effects.

### Monitoring disease response and detecting recurrence

In addition to assessing current disease status, hematocrit metrics are valuable for monitoring disease response and detecting potential recurrence[37]. During treatment, regular measurement of hematocrit can help evaluate how well the therapy is working and whether the disease is under control. After the completion of treatment, continued monitoring of hematocrit levels can be part of a surveillance strategy to detect early signs of disease recurrence. A sudden change in hematocrit levels after a period of stability might indicate the return of the disease or the development of new complications, prompting further diagnostic testing and intervention.

### Evaluating the impact of targeted therapies

Emerging targeted therapies for breast cancer, such as HER2 inhibitors and CDK4/6 inhibitors, have specific effects on tumor cells and can influence hematologic parameters. Hematocrit metrics can be used to evaluate the impact of these therapies on the patient’s blood health. For example, targeted therapies that affect RBC production or bone marrow function might lead to changes in hematocrit levels, which can be monitored to assess treatment efficacy and manage side effects. This evaluation helps ensure that targeted therapies are delivering the intended therapeutic benefits while minimizing adverse effects.

### Assessing nutritional status and cachexia

Breast cancer patients are at risk for nutritional deficiencies and cachexia, a syndrome characterized by severe weight loss and muscle wasting[38]. Nutritional deficiencies can lead to anemia and altered hematocrit levels. By tracking hematocrit metrics, clinicians can gain insights into the patient’s nutritional status and address deficiencies that might be contributing to anemia. For instance, low hematocrit levels may prompt a nutritional assessment to identify and correct deficiencies in essential nutrients like iron, vitamin B12, and folate.

### Evaluating the effectiveness of supportive therapies

Supportive therapies, such as erythropoiesis-stimulating agents and blood transfusions, are often used to manage anemia in breast cancer patients[39]. Hematocrit metrics are instrumental in evaluating the effectiveness of these supportive treatments. Regular monitoring of hematocrit levels can help determine if the administered therapies are achieving the desired outcomes, such as increasing RBC counts and improving patient symptoms. This assessment guides adjustments in supportive care strategies to optimize patient health and QoL.

### Enhancing personalized cancer care

The integration of hematocrit metrics into breast cancer care supports a more personalized approach to treatment[40]. By providing a dynamic and noninvasive measure of disease status, hematocrit metrics enable clinicians to tailor treatment plans based on real-time data. Personalized cancer care involves adjusting therapies according to individual patient responses, and hematocrit metrics can be a valuable tool in this process. By incorporating these metrics into routine care, clinicians can offer more precise and effective management strategies tailored to each patient’s unique needs.

## Recent advances in hematocrit-based biomarkers in breast cancer surveillance

In recent years, hematocrit-based biomarkers have garnered increasing attention as a promising tool for breast cancer surveillance. Hematocrit, a measure of the proportion of RBCs in blood, reflects various physiological and pathological conditions, including the body’s response to cancer. Innovations in hematology and a deeper understanding of tumor biology have led to significant advances in the use of hematocrit-based biomarkers for monitoring breast cancer. This section discusses recent developments in this area and their potential impact on clinical practice[41].

### Understanding hematocrit as an indicator of tumor-associated inflammation

Hematocrit levels can be influenced by systemic inflammation, which is a hallmark of cancer progression. Research has shown that breast cancer tumors, particularly those with a higher degree of malignancy, can induce a pro-inflammatory state in the body. This inflammation can lead to changes in RBC production, impacting hematocrit levels. Recent studies have emphasized the role of hematocrit as a potential indirect marker of tumor-associated inflammation, which could be leveraged to monitor the disease’s systemic impact. Studies have found a correlation between low hematocrit levels and the presence of advanced breast cancer. Tumors may cause alterations in bone marrow function, contributing to anemia, a common complication of cancer. Furthermore, systemic inflammation related to breast cancer may be captured by hematocrit fluctuations, offering a real-time indicator of tumor burden and progression[9].

### Hematocrit as a marker of anemia in breast cancer patients

Anemia is a frequent consequence of breast cancer, especially in patients undergoing chemotherapy or radiation therapy. Recent research has demonstrated that hematocrit levels, alongside hemoglobin concentrations, can be used to assess the severity of cancer-related anemia. Anemia not only reflects the progression of cancer but also has significant prognostic implications. Hematocrit-based biomarkers are now being explored for their potential to gauge both treatment-related anemia and its correlation with treatment efficacy. A study published in *Breast Cancer Research and Treatment* found that hematocrit, as part of a complete blood count, can provide early indications of anemia before it becomes clinically significant, allowing for preemptive management. Tracking changes in hematocrit can also help monitor the effects of chemotherapy, providing an early indication of how a patient is responding to treatment, particularly in those undergoing aggressive regimens that may affect bone marrow function^[42,43]^.

### Hematocrit and tumor oxygenation

Tumor oxygenation is a critical factor in breast cancer progression and response to therapies. Hypoxia, or low oxygen levels in the TME, plays a significant role in promoting tumor aggressiveness, metastasis, and resistance to treatment. Hematocrit has recently been studied as a potential marker for tumor oxygenation, as it is indirectly related to oxygen-carrying capacity in the bloodstream. Research has shown that low hematocrit levels could reflect poor tumor oxygenation, as anemic patients may have less oxygen available for tissues, including tumors. Advances in noninvasive imaging technologies, such as functional MRI and PET scans, have further supported the potential of hematocrit as a marker for tumor hypoxia. Hematocrit levels, combined with oxygenation imaging, could provide a more comprehensive understanding of tumor biology and guide treatment strategies aimed at normalizing oxygen levels within tumors[44].

### Integration of hematocrit with other biomarkers for early detection and surveillance

A significant recent advance is the integration of hematocrit-based metrics with other biomarkers and imaging techniques for early detection and ongoing surveillance of breast cancer. The combination of hematocrit with molecular biomarkers, such as circulating tumor DNA (ctDNA), tumor-associated antigens, or microRNAs, has shown promise in enhancing diagnostic sensitivity and specificity. Several studies have demonstrated that combining hematocrit levels with biomarkers like HER2 status, ER, and progesterone receptor status improves the ability to track disease progression and response to treatment. This multi-biomarker approach enables a more nuanced, dynamic understanding of breast cancer biology. In a study examining breast cancer recurrence, researchers found that changes in hematocrit levels, when paired with ctDNA testing, allowed for earlier detection of relapse than using either marker alone. This approach could potentially reduce the need for more invasive diagnostic procedures, such as biopsies^[45,46]^.

### POC hematocrit testing for breast cancer monitoring

Advances in hematology technologies have made it possible to measure hematocrit levels at the point of care, offering a convenient and efficient way to monitor patients during treatment. The development of portable, easy-to-use devices for blood testing has the potential to revolutionize breast cancer monitoring, particularly in low-resource settings where access to specialized diagnostic tools may be limited. POC devices that rapidly assess hematocrit levels could provide clinicians with real-time data, enabling them to make quicker decisions about treatment adjustments, such as when to initiate blood transfusions or adjust chemotherapy regimens. These devices, in combination with digital health platforms that track patient data over time, could facilitate continuous monitoring, empowering patients and providers to manage breast cancer in a more proactive and personalized manner^[47,48]^.

### Hematocrit-based metrics for predicting treatment response

Recent studies have indicated that changes in hematocrit levels during treatment could serve as a predictor of treatment efficacy. Monitoring fluctuations in hematocrit may provide insight into how well a patient is responding to chemotherapy, immunotherapy, or targeted treatments. By analyzing hematocrit trends alongside other biomarkers, clinicians can identify patients who may require a change in their therapeutic regimen. A study published in *Cancer Chemotherapy and Pharmacology* showed that patients with stable or rising hematocrit levels during chemotherapy had better treatment responses compared to those with decreasing hematocrit levels, who were at higher risk for treatment failure. Hematocrit changes have been proposed as part of an early-warning system to detect resistance to treatment. Monitoring hematocrit levels could allow for timely intervention, optimizing the chances of successful treatment outcomes^[49,50]^.

## Challenges and future directions in breast cancer monitoring

Despite the promising potential of emerging biomarkers, including hematocrit-based metrics, in breast cancer monitoring, several challenges remain in the path toward their widespread clinical adoption. Addressing these challenges and exploring future directions for research and clinical practice is essential for advancing breast cancer care.

### Challenges

1. Confounding factors and variability: Hematocrit levels can be influenced by a variety of non-cancer-related factors, such as hydration status, altitude, other comorbid conditions (e.g. anemia, cardiovascular diseases), or even medication use. These confounders can complicate the interpretation of hematocrit data in the context of breast cancer. For instance, chemotherapy or radiation therapy can induce anemia or alter hematocrit levels independent of tumor activity, making it difficult to distinguish between treatment-related effects and disease progression[51].
2. Lack of standardization: The measurement and interpretation of hematocrit levels can vary depending on the laboratory, the methods used, and the population being studied. For hematocrit-based metrics to be a reliable tool in clinical practice, it is crucial to establish standardized protocols for measurement, threshold values for various stages of disease, and consistent methodologies for integrating hematocrit data with other diagnostic markers. The absence of such standards can lead to inconsistencies in clinical decision-making and limit the generalizability of findings[52].
3. Tumor heterogeneity: Breast cancer is a heterogeneous disease with multiple subtypes, including hormone receptor-positive, HER2-positive, and triple-negative breast cancer. Each subtype behaves differently, responds to treatment in distinct ways, and may influence hematocrit levels differently. The TME, immune response, and systemic effects of cancer vary greatly across patients, and this heterogeneity can make it difficult to use hematocrit metrics in a one-size-fits-all manner. Further research is needed to understand how hematocrit correlates with specific subtypes and clinical outcomes[53].
4. Integration with other biomarkers: While hematocrit-based metrics hold promise, relying on them alone is unlikely to provide a complete picture of disease status. Breast cancer is complex and multifactorial, and using hematocrit in isolation may overlook other critical markers of disease progression, such as tumor size, genetic mutations, and receptor status. For hematocrit metrics to be truly valuable, they must be integrated with other biomarkers, imaging techniques, and clinical assessments. Developing algorithms and diagnostic frameworks that combine hematocrit with other markers will be essential to ensure its effectiveness in clinical settings[54].
5. Monitoring TME and response: Tumor hypoxia, an essential factor in cancer progression, plays a significant role in breast cancer’s response to therapies. Hematocrit can provide an indirect measure of tumor oxygenation, but its role in evaluating TME dynamics is still not fully understood. Future research should explore the relationship between hematocrit fluctuations and specific tumor characteristics (e.g. size, stage, vascularization) to determine how it can best inform treatment decisions[51].

## Future directions

1. Personalized and precision medicine: The future of breast cancer monitoring lies in personalized and precision medicine, where treatment is tailored to the unique characteristics of the patient’s cancer. Hematocrit-based metrics could play a key role in this approach by providing dynamic insights into how a patient’s body is responding to therapy. By combining hematocrit levels with genetic and molecular profiling of tumors, clinicians could create more individualized treatment regimens that are optimized for each patient’s specific needs. Longitudinal monitoring of hematocrit, alongside other biomarkers, may help guide treatment adjustments in real time, improving overall efficacy and minimizing unnecessary side effects[52].
2. Combination with other diagnostic tools: While hematocrit-based metrics are promising, they should not be viewed as a replacement for existing breast cancer monitoring tools but rather as a complementary addition. Future research should focus on developing multimodal diagnostic approaches that combine hematocrit with imaging, tissue biomarkers, and advanced genetic profiling. For example, integrating hematocrit metrics with ctDNA or imaging techniques such as functional MRI could provide a more comprehensive understanding of tumor dynamics and treatment response. This holistic approach could allow for more precise monitoring of disease progression, response to therapy, and early detection of recurrence[53].
3. Advanced hematology technologies: The future of hematocrit-based metrics may also involve advances in hematology technologies, such as high-throughput blood tests, that could enable more frequent and accurate monitoring of hematocrit levels. Advances in POC testing and portable diagnostic devices could allow for regular, real-time monitoring of hematocrit levels in breast cancer patients. This would offer a noninvasive, cost-effective, and timely means of tracking disease progression, particularly for patients in remission or undergoing long-term treatment[54].
4. TME and hypoxia-research: Further research into the relationship between hematocrit and the TME is essential for understanding how variations in hematocrit levels correlate with tumor hypoxia, angiogenesis, and response to therapies. The ability to monitor the tumor’s oxygenation status through hematocrit could guide clinicians in determining the most effective therapeutic strategies, including those targeting the tumor vasculature, hypoxic regions, or immunotherapy.
5. Longitudinal studies and clinical trials: More extensive longitudinal studies and clinical trials are needed to validate hematocrit-based metrics as reliable tools for monitoring breast cancer. Such studies should include diverse patient populations, varying stages of disease, and different breast cancer subtypes. Additionally, trials that combine hematocrit metrics with other biomarkers, imaging modalities, and therapeutic interventions will help elucidate their role in clinical practice.
6. AI and ML: The application of AI and ML algorithms to hematocrit-based data has the potential to further revolutionize breast cancer monitoring. AI and ML can analyze complex data sets, identify patterns, and predict patient outcomes based on hematocrit fluctuations in conjunction with other clinical data. These technologies could aid in early detection, treatment planning, and recurrence prediction, ultimately leading to more precise and personalized breast cancer care.

Table 1 shows a summary table showing Revolutionizing Breast Cancer Monitoring: Emerging Hematocrit-Based Metrics (provided by the authors).

**Table 1:** **A summary table showing** revolutionizing breast cancer monitoring: emerging hematocrit-based metrics – a narrative review

**Category**	**Key points**
Objective	To evaluate the role of hematocrit-based biomarkers in breast cancer surveillance, exploring their potential to detect tumor-associated inflammation, monitor anemia, assess tumor oxygenation, and predict treatment responses
Literature search strategy	Databases searched: PubMed, Google Scholar, Scopus, Web of Science, Embase. Keywords: “Hematocrit,” “Breast cancer,” “Surveillance,” “Anemia,” “Biomarkers,” “Tumor hypoxia,” “Treatment response
Inclusion criteria	Studies focusing on breast cancer surveillance using hematocrit as a biomarker; clinical trials, systematic reviews, meta-analyses, and observational studies
Exclusion criteria	Studies not related to hematocrit or breast cancer; animal studies; non-English publications; articles without full-text access
Study selection process	Initial screening of titles and abstracts; full-text review for methodological quality; 45 studies included
Key themes in data synthesis	Hematocrit as a marker of tumor-associated inflammation in breast cancer. Role in monitoring cancer-related anemia. Association with tumor oxygenation and implications for therapies. Point-of-care hematocrit testing for real-time surveillance. Combining hematocrit with other biomarkers for enhanced monitoring
Quality assessment	Studies evaluated based on design, sample size, and risk of bias using tools such as Cochrane’s Risk of Bias and the Newcastle-Ottawa Scale for cohort studies
Recent advances	Emerging research on integrating hematocrit with other markers, use in treatment response monitoring, innovations in point-of-care hematocrit testing, and its potential in assessing tumor oxygenation
Challenges identified	Variability in study designs and methodologies. Limited sample sizes in some studies. Publication bias in reporting positive findings. Challenges in standardizing hematocrit measurement techniques
Future directions	Larger, more diverse clinical trials with standardized methodologies. Integration of hematocrit with other biomarkers for more comprehensive surveillance. Development of more accessible point-of-care testing for real-time monitoring. Exploration of hematocrit’s role in personalized cancer treatments and precision medicine
Conclusion	Hematocrit-based biomarkers show promise for breast cancer surveillance, providing insights into tumor-associated inflammation, anemia, and treatment response. However, further research and improved diagnostic integration are needed to fully harness their potential in clinical practice

## Conclusion

Hematocrit-based metrics represent a promising and innovative approach to breast cancer monitoring, offering a dynamic, noninvasive, and cost-effective tool to enhance patient care. These metrics provide valuable insights into a variety of aspects of breast cancer, including tumor-induced inflammation, anemia, tumor oxygenation, and treatment response. By tracking hematocrit levels over time, clinicians can gain real-time information about disease progression, evaluate the effectiveness of therapies, and detect recurrence at an early stage.

## Data Availability

Not applicable.
